# Rapid Creation of an Online Discussion Space (r/nipah) During a Serious Disease Outbreak: Observational Study

**DOI:** 10.2196/13753

**Published:** 2019-11-13

**Authors:** Jennifer Cole, Saphia Ezziane, Chris Watkins

**Affiliations:** 1 Royal Holloway University of London Egham United Kingdom

**Keywords:** information science, internet, disease outbreaks, public health, behavior, communication

## Abstract

**Background:**

During health emergencies, the people within affected communities ask many questions at a time when professional medics and health agencies are overstretched and struggling to cope. Our previous research has shown that, during the 2014-2015 West Africa Ebola crisis, volunteer-moderated online discussion forums were able to provide peer-to-peer reliable, trustworthy, and well-managed information. We speculated that with the right mix of epistemic and experiential knowledge, such a discussion forum could be set up rapidly during a future serious disease outbreak.

**Objective:**

The aim of this study was to set up a peer-to-peer health information exchange forum within the shortest time possible after the emergence of a real outbreak of a serious infectious disease. An outbreak of Nipah virus in Kerala, India, in May 2018 provided the opportunity to test our theories.

**Methods:**

We initiated a Nipah virus discussion forum on the platform Reddit, recruiting volunteer moderators from within the existing Reddit community. This facilitated posts and comments to the forum from genuine Reddit users. We gathered and analyzed data on the number of posts, comments, page views, and subscribers during the period of May 24 to June 23, 2018, by using the data analysis tools embedded in the Reddit platform.

**Results:**

We were able to set up a functioning health information exchange platform by May 24, 2018, within two weeks of the index case and one week of the official World Health Organization verification of a Nipah virus outbreak. Over the following five weeks, the forum received a steady flow of traffic including posts (36) and comments (21) submitted, page views (840), and subscribers (33). On the busiest day, 368 page views were recorded. The forum provided information in the languages spoken in the outbreak region as well as in English on how the virus spreads, symptoms of the disease, and how to take measures to avoid contracting it. Information on government helpline numbers and frequently asked questions was also provided to the community at risk.

**Conclusions:**

The delivery of a fully functional discussion forum within a short space of time during an actual health emergency demonstrates that our suggestion is fully practical. Our theory that Reddit could provide a suitable platform to host such a forum was upheld. This offers great potential for public health communication during future serious disease outbreaks.

## Introduction

### Background

In a public health emergency when health care systems may be overwhelmed, the ability of affected communities to crowdsource reliable information could have a significant impact on the extent to which a disease outbreak might be contained or its spread slowed [1-3]. Funk et al [4] have theorized that behavioral changes might influence the spread of disease, and Funk and Jansen [5] have modeled the impact of peer influence on disease spread. Cole [6] has investigated how reliable, trustworthy, and timely public health information can be exchanged over peer-to-peer platforms, including in the event of a public health emergency, and how this might be best achieved by using platforms that are already widely used by the affected population—in particular, the already existing and highly popular information sharing platform Reddit. Her case study on the use of Reddit during the 2014-2016 Ebola virus outbreak in the West African countries of Sierra Leone, Liberia, and Guinea [7] suggests that such forums can provide a useful resource during disease outbreaks.

### Near at Risk, Far at Risk, Real at Risk

Cole and Watkins [8] have identified three different stages through which people experience disease outbreaks: Real at Risk, Near at Risk, and Far at Risk. In each stage, how people seek out information and the type of information they require differs. Real at Risk information seekers are defined as being in close physical proximity to a person displaying symptoms or known to be infected. Near at Risk information seekers live in a region where cases have been recorded in locations they are likely to visit, requiring behavior modifications that will reduce their likelihood of becoming infected. Far at Risk information seekers are geographically distanced from outbreaks and in no real danger of contracting the virus. Closeness can be defined not only by geographical proximity to cases of the disease but also by intimacy and socioeconomic similarity to those affected [7-9]. Participation in online forums can lead participants to perceive themselves as being in a higher risk category than is actually the case [7-9], opening up interesting possibilities for spreading public health information and encouraging risk avoidance behavior to be adopted ahead of the actual arrival of a disease outbreak.

### From Passive Absorption of Facts to Active Questioning

During the Far at Risk phase of the 2014-2016 Ebola outbreak, health information seekers were content to passively collect facts, statistics, and scientific information relating to disease characteristics and spread. During the Near at Risk and Real at Risk phases, however, interaction, discussion, and advice tailored to personal circumstances became increasingly important. The community came together in online forums to answer each other’s questions and provide advice, particularly during the latter phases of the outbreak when an increasing number of people had the experience of living with the disease [7].

When information on how to protect oneself from infection, how to recognize symptoms, and how to treat loved ones at home is provided through peer-to-peer online communities rather than through professional authorities, credibility of the information provided becomes vital [10,11]. Advice must be accurate if it is to be not only distributed but also acted on [6]. In online forums, the community can collaborate by upvoting material as well as posting it, making particular posts not only more visible but also adding to their credibility: users are more likely to trust information that other users have shown they also trust and value [7,12].

### Credibility of Information

On the subreddit r/ebola, community collaboration allowed for credible and accurate information to be shared among those experiencing the crisis firsthand, while also providing factual information for those Far at Risk [8]. The quality of information presented on subreddits depended, however, not only on contributors who had expertise in the subject matter, but also on expert moderators who managed the forums and regulated the posts. These expert moderators use technical skills to code the subreddit and set automoderation tools that reduce the time burden on the human moderators by, for example, automatically removing posts that contain racist or sexist terms or profanity or those from media sources known to be sensationalist or biased. Equally important is experiential knowledge of the platform that enables a forum to scale up quickly in times of high traffic and recruit quickly new moderators who have specific skills, experience, and knowledge. For example, moderators of other forums on the same platform may have knowledge of local languages spoken in the affected region or of local conditions and facilities. Experiential knowledge of the platform is vital for knowing how to seek out individuals with these skills.

### Lessons From Ebola

The Ebola virus public health emergency of international concern (PHEIC) of 2014-2016 led to high rates of morbidity and mortality that imposed a severe economic burden on West Africa but also provided a key insight into the information requirements of those in the midst of an epidemic and how a peer-to-peer information platform could address this. It showed that there are three linked requirements needed to set up and manage a forum: subject matter expertise, experience with the platform chosen for use, and technical ability, all of which are essential to enable a discussion forum capable of hosting thousands of users to be set up quickly in the wake of an unexpected and rapid-onset outbreak of infectious disease [7].

Existing sites such as Reddit that are widely used and already familiar to many users have the potential to become highly trusted platforms for sharing vital medical information during a pandemic, epidemic, or outbreak. They can provide information that is accurate and well regulated [6,7]. Prior to 2018, this hypothesis had not been tested during a real-life outbreak of a serious infectious disease. We had considered creating a subreddit dedicated to a disease outbreak in the hope of validating our theory but needed an actual disease outbreak to occur in order to test it in vivo.

### Kerala Nipah Virus Outbreak

The opportunity to create such a subreddit arose in May 2018 [13], when an outbreak of Nipah virus (NiV), a viral infection causing severe flu-like symptoms with a case fatality rate of 50% to 75%, occurred in the Indian state of Kerala.

On May 19, 2018, the World Health Organization (WHO) confirmed that three people had died in Kozhikode District, Kerala State, India, due to NiV [14]. NiV is a zoonotic disease that can be transmitted to humans from animals such as pigs and bats (fruit bats of the Pteropodidae family are widely accepted to be the natural host), through direct human-to-human contact, or through consumption of contaminated foods, in particular fruits and palm sap contaminated with bat saliva or urine [15,16]. At the time of the Kerala outbreak, there was no vaccine for the disease, which could only be treated by supportive care [17].

To validate our hypothesis that sites such as Reddit can be a stable platform through which to share useful information (including medical advice, likely sources of infection, routes of primary and secondary transmission, and effective treatment options) with affected communities in the midst of a public health emergency, we aimed to build a subreddit dedicated to discussing the outbreak, from scratch, in as short a time as possible that could serve a genuinely at-risk community.

### Reddit Use in Kerala

For our experiment to work, the population affected by the outbreak needed to be using Reddit prior to the outbreak. Kerala has a population of 34 million [16] and an above average level of affluence for India [18]; at the time, average income in the region was Rs 59,000 (US $850) per annum compared with Rs 38,900 (US $550) for India as a whole. Educational attainment is also higher than in surrounding regions; the literacy rate for Kerala was 94% compared with a national average of 74%. A subreddit dedicated to the region, r/Kerala, has been active since March 2008 [19]. At the time of the NiV outbreak, this forum had just over 4000 subscribers. Observation of the discussions taking place on the site suggested that most users were based in Kerala, and the forum serves the community rather than being an information site for visitors or tourists.

The NiV outbreak, therefore, offered an ideal opportunity to create an online health information forum on an existing and widely used platform that could potentially be taken up and used by a population Near at Risk to a serious disease outbreak.

## Methods

### Building a Forum

Our previous research [6-8] has indicated that forums for high-quality peer-to-peer information exchange during disease outbreaks work well when existing popular platforms are used and the forum moderators have experience in both the subject under discussion and the platform on which the discussion is hosted. We therefore aimed to build a suitable forum on an appropriate platform and recruit a moderator team to run it.

We chose to build the forum on Reddit, a news aggregator site that also hosts discussion forums. It is one of the world’s 20 most popular websites [20], allowing users to post content, comment on content, and vote on both posts and comments made by other users. Content is socially curated and promoted by site members through voting [21]. Reddit is composed of hundreds of thousands of individual subreddits—forums on specific topics—all of which share information and host discussions to which any reddit user is free to contribute. Our previous research has shown that Reddit can host huge discussions and is capable of rapid growth [7].

Each subreddit is monitored and managed by volunteer moderators who are able to set rules and remove posts and comments that are deemed inappropriate, offensive, or inaccurate (for example, those that contain information that is factually incorrect, use racist or sexist language or profanities, or are aggressive), resulting in an online space where useful and interesting information can be shared among users. Further research has shown that while the quality of the information on such forums is variable, on well-managed forums it can be scientifically accurate and in line with accepted medical practice [6]. When doctors were asked to rate the usefulness and accuracy of comments and posts, the ratings they gave the information validated the Reddit community’s perception of which contained the better information [7]. As such, we believe that Reddit has many characteristics conducive to ensuring the promotion of reliable, trustworthy, and high-quality information, including its voting structure, volunteer moderators, and complex system of trust markers [6,7].

The suggestion to set up r/nipah was initially made by an experienced reddit moderator, known by the username u/IIWIIM8, who had been interviewed during our previous r/ebola study [7] and remained in contact with the research team. This moderator suggested that a forum dedicated to sharing valuable content about events and the infection itself (such as symptoms and treatments) and responding to common queries through frequently asked questions (FAQs) would be beneficial to the affected community. The subreddit r/nipah was set up through the Reddit platform on May 24, 2018, by u/JenniferColeRHUK (one of the authors of this paper). The user u/IIWIIM8 was invited to become a moderator and provide technical and experiential expertise, with u/JenniferColeRHUK proving subject matter expertise if and when appropriate.

### Recruiting a Moderator Team

Once the subreddit had been initiated, we next needed to recruit an effective group of moderators to help keep the forum running. Our previous study [7] had identified three distinct moderation tasks that needed to be covered by a moderation team (or a single moderator): subject matter expertise, technical expertise, and experience in Reddit’s norms and structure. We were able to access and draw on all of these through the Reddit community and construct an effective and efficient moderator team within a reasonably short time frame.

We set out to recruit a team of moderators that conformed to the skillset identified above and the group dynamic identified as most beneficial to enabling the emergence of collective intelligence [22], a form of crowd wisdom greater than the sum of its parts. Through a post made to r/CSShelp [23] we recruited u/nortonism, a CSS programmer willing to help with formatting the forum, in particular to help with setting up the AutoModerator, an inbuilt function that can automatically remove information posted by users that is insensitive (eg, racist language suggesting that lack of personal hygiene is responsible for the outbreak) or inaccurate (eg, comments that might suggest the virus is airborne if this is not the case). We invited u/rodomontadeferrago, a Reddit user who was posting useful information on r/Kerala, including a well-received FAQ, to provide subject matter expertise and later become a moderator. The additional moderators listed on the forum are the authors of this paper (u/Snowflake1000, u/breezehair, and u/roses1997) and a bot (u/BotBust).

We also approached posters who were posting information on the NiV outbreak, including information on its transmission and government responses to the outbreak, in r/India [24] and r/Kerala [25], directing them to the r/nipah subreddit. We asked them to cross-post (enabling content they posted to appear on more than one subreddit) and conferred Approved Submitters status on those providing high-quality information.

### Data Collection

Data from the subreddit were collected, added to an Excel spreadsheet (Microsoft Corp), and analyzed (Multimedia Appendix 1). Data collection included the number of posts and details of each post by username, time and date, number of comments received, points received (average of upvotes and downvotes), user karma (posting history and rating), and whether the post was cross-posted.

## Results

### Platform Construction and Management

Drawing on Cole’s [6,7] previous experience with Reddit (gained during the Ebola PHEIC of 2014-2016), a functioning health information exchange platform, the subreddit r/nipah, was set up on May 24, 2018. The first post, titled “Welcome to r/nipah” [26], introduced the subreddit as a place to “share reputable news items on Nipah outbreaks, factually correct scientific information about Nipah, and advice on what to do if you are concerned about outbreaks.” The forum encouraged discussions between those affected and medical professionals in the local area or more distant.

The forum was constructed on the existing Reddit platform within 2 weeks of the index case and just 5 days after the official WHO verification of a NiV outbreak on May 19, 2018, following three deaths in Kerala [13]. This was within the first 10 days after the outbreak was announced, the prime time frame required for containment [1].

During the first week of the forum’s existence, 18 posts were contributed by moderators and 5 spontaneous users from the Reddit community who simply wanted to share information and were not part of the research team, following requests for contributions on r/Kerala and r/India. Early posts included links to NiV fact sheets produced by the WHO and the US Centers for Disease Control and Prevention (CDC), news items on crematorium staff shunning NiV victim’s bodies, and advice on food safety and personal hygiene. After a Reddit user asked whether they should seek medical diagnosis for a mild fever [27], information was provided on government helpline numbers in India. Links were also posted to responsible professional media coverage from agencies such as the Times of India. The forum had already been running for 4 days when the spread of the virus appeared to worsen: 15 people in Kerala were infected by May 28, 2018 [13].

Over the following 4 weeks, until June 23, 2018, when the outbreak was considered to be over [28], the forum received 36 posts and 840 page views (including 368 on the forum’s busiest day, May 26, 2018). Posts linked to news items from reputable international sources such as WHO, Stanford University, and the Times of India giving scientific information about how the virus spread and how the outbreak was being managed by the local authorities. The forum provided information in local languages as well as English. Permanent links to government helpline numbers were posted and disease-specific FAQs—lists of questions frequently asked by posters, with answers provided—were developed.

The users of the forum expressed views that located them in the affected regions of India (eg, by stating where they lived or worked) and thus indicated that participants did include some Near at Risk candidates, although it was not possible to determine the location of all forum users.

### Moderator Team

We recruited a diverse set of moderators who were male and female, aged 21 to more than 50 years, based in America and the United Kingdom with support from approved posters in India. The moderators displayed a diverse skillset covering technical expertise, subject matter expertise, and experience using Reddit. Two of this paper’s authors (JC and CW) are sufficiently experienced Reddit users and moderators to be considered a genuine part of the moderation team; SE was a nonparticipant observer. Reddit users u/nortonism and u/IIWIIM8 (neither had an academic affiliation) ensured that only relevant posts and comments appeared on the subreddit, coded the flair categorizations for the posts, and organized the layout of the subreddit. A further moderator recruited from the Reddit community, u/rodomontadeferrago, provided subject matter expertise. The moderation team were all Far at Risk [8], geographically distributed between the United Kingdom and the United States. With the moderators regulating site content, the site was left open for Reddit users who were either experiencing or interested in the crisis to post material.

### Posts Made to the Subreddit r/nipah

Across the 31-day time period between May 24, when the forum was set up, and June 23, when the forum users and local Kerala newspapers considered the outbreak to be over [29] (although the official WHO declaration was not made until 6 weeks later [28]), 36 posts were made to r/nipah. These consisted of 29 link posts (links to content posted on external websites such as news media, WHO, or CDC) and 7 self-posts (generally questions or comments that do not link to content hosted elsewhere) of which 3 were FAQs. The site gained 33 subscribers during this period. Self-posts included FAQs to help those who were Real at Risk assess the crisis and a medical query from someone in fear of having caught the illness. An external user also shared their suggestions for how to improve the subreddit site, such as a crackdown on jokes that some users might find inappropriate. When information was posted in Hindi, which none of the moderators spoke and whose quality they were therefore unable to verify, moderators sent a personal message through the Reddit internal mail system to one of the posters known to be based in India, u/Valarauko, asking for translation. A full translation was provided and posted on the site for the benefit of other users.

The 36 posts received an average score of 3 points per post (range 1-6) from the community, indicating an average ratio of 3 upvotes to every downvote received with a range of 60% to 100% upvoting (specific numbers of upvotes-to-downvotes are not available). A total of 113 points were recorded across all posts.

The forum received a reasonable amount of community interaction during the outbreak, including 21 comments made against the posts and 12 cross-posts to or from other subreddits including r/India, r/generalsciences, and r/medicine. Discussions under the top-level posts addressed the validity of articles posted on r/nipah, r/sciences, r/infectiousdiseases, r/Kerala, and r/EcoInternet.

Once the NiV outbreak was announced to have been fully contained in Kerala, on June 9, 2018, posting activity slowed down on the site. The final active post made within the lifetime of the outbreak, titled “Kerala bids musical farewell to Nipah virus—r/Kerala” [29], was posted on June 23, 2018. This was followed by a disclaimer post [30] explaining that the subreddit had been set up as part of an academic research project and inviting any other researchers to contact us to share data and findings.

## Discussion

### Principal Findings

The r/nipah subreddit does seem to have achieved its ultimate goal of creating a safe space for information exchange where people could refer to information relating to the NiV outbreak, make queries, and receive reputable responses. As far as we can ascertain, the content on the subreddit contained high-quality information, with no conspiracy theory posts or scaremongering. Genuine concerns were expressed and answered with useful information and advice. Very quickly, we had created the basis of what could potentially become a very useful public information platform.

In comparison with many subreddits, r/nipah attracted only a modest number of subscribers (33), however. This is likely to be due to the small-scale nature of outbreak and the fact that authorities handled the situation well. With the outbreak under control, there was little need for an unofficial information forum. Had the outbreak spread and the situation become more serious, we believe we had put in place the necessary platform architecture, information, and expertise needed to run the forum; however, there was insufficient opportunity to test this fully or assess the extent to which the forum made a measurable contribution to the management, progression, or containment of the outbreak.

Nonetheless, we feel that the Kerala NiV outbreak of 2018 provided an opportunity to study the efficacy of health solutions offered via Reddit. It allowed us to study a real outbreak in real time that occurred in an area where many people already used the forum, evidenced by the existence of both r/Kerala, a local community forum used by people living in Kerala, and r/India. This was a major difference from the Ebola outbreak studied in our previous research [7] where internet penetration in the affected region was low, and people did not, by and large, post on Reddit. The r/ebola community was almost entirely Far at Risk, while Real at Risk nongovernmental organization workers interviewed during the same study were not widely using Reddit. The r/Kerala community was, by contrast, Near at Risk with a realistic likelihood of a transition to Real at Risk and did use Reddit.

The heavier ratio of link posts to self-posts (4:1) seen on r/nipah is consistent with the pattern that we would have expected from our previous research [7,8], which is that when cases and perceived risk are low, information seekers are more interested in facts and statistics and are happy to passively receive information from the media and official sources but do not necessarily want to interact or discuss that information through two-way dialogues.

### Spread of Conspiracy Theories

Unlike on other disease outbreak subreddits including r/ebola and r/zika, no conspiracy or openly negative posts were made on r/nipah that could have hindered its ability to maintain a safe space in which to discuss the events. This was possibly because there were no existing antigovernment or antitechnology narratives on which conspiracy theorists felt the need to jump; conspiracy theories on other forums tend to rely on preexisting narratives (eg, that the Sierra Leone government was prone to kill off voters in regions held by the opposition party, seen during the Ebola outbreak, or the narrative that genetically modified crops were responsible for the Zika virus outbreaks in South America [7]). This may be because, in contrast to the 2014-2016 Ebola outbreak, the Kerala NiV virus outbreak was small in terms of the geographic area affected and number of cases recorded and thus did not attract disruptive attention. Another reason might be that as the outbreak appeared to be handled well by the national government and health care system, there was little room for credible criticism.

By distributing tasks across a moderator team, with some taking on dedicated roles such as programming the AutoModerator function, pressure was taken off the team during a period when the site was likely to experience rapidly increasing traffic.

### Location of Posters

One final observation of interest is that the profiles of users who posted and their activity on other subreddits suggests that more of them were Far at Risk than Near at Risk or Real at Risk. Even those who posted from within India did not seem to be within close proximity to Kerala, although they did express genuine concerns. This is, however, an assumption based on user activity observed: we cannot confirm the actual location of each user. An interesting avenue of future research would be an analysis of the geographic location of users, which may enable us to better understand the audience the subreddit attracted: did it truly serve those who were Real at Risk/Near at Risk of contracting NiV or only those who were Far at Risk but interested in the outbreak? These data would be crucial in helping to develop a site that would fully prove our theory. However, we also suggest that providing information to people ahead of their actual exposure to the virus, enabling them to learn about it, prepare for it, and consider how they might need to modify their behavior in the event of a closer outbreak, is unlikely to have any disadvantages and may help communities to remain calm during an escalating epidemic.

The results we obtained indicate a measure of success: a fully functional discussion forum was constructed swiftly within a short space of time, an effective moderator team was recruited, and although the number of subscribers and posts was small, the information provided was high quality. The self-posts and FAQs in particular stand as a good example of how the community came together on this platform. This was all achieved during an actual health emergency, proving our theory that such a delivery is practical and fully achievable. We believe that these results validate our idea that Reddit can be a suitable platform where those experiencing or interested in serious disease outbreaks can come together to share valuable information and advice regarding the crisis.

This research offers great potential for public health communication during future serious disease outbreaks, particularly if such a discussion platform could be supported by or linked to an existing trusted health brand such as ProMED or WebMD and used in conjunction with notifications of disease outbreaks to provide information on how best to react to known and expected events. Public communication at scale is completely feasible; well-moderated subreddits manage discussions with contributions from tens of thousands of users and tens of millions of readers.

### Limitations

The study is limited by the resources available to the researchers and the information available on the Reddit users. We were not able to confirm, for example, the geographic location of the posters or of the Reddit users responsible for the page views and so cannot determine whether the majority were Far at Risk, Near at Risk, or Real at Risk. Access to such information in future studies would be advantageous.

The study was also limited by the low number of cases of NiV infections, which did not overstretch local health care systems. Despite the mortality rate of the virus being significantly high, with a 74.5% average case fatality rate [31], the Indian government was able to contain the outbreak rapidly. It is therefore somewhat unrealistic to compare r/nipah and the NiV outbreak with r/ebola, as the 2014-2016 West African Ebola outbreak lasted for more than 2 years and affected a much larger global area. The situation also differed in that the NiV outbreak took place in a middle-income country where there was much higher access to and use of the internet.

### Comparison With Prior Work

This work draws largely on our own previous research into the potential use of peer-to-peer, self-regulated discussion forums (particularly those hosted by Reddit) during a PHEIC featuring a serious infectious disease with limited treatment options [6-8]. We are not aware of any other research teams that are currently examining the potential use of such forums during similar events or other public health emergencies and consider our approach to be unique.

### Conclusions

We had previously theorized that we could use an online social networking platform such as Reddit to be a valuable knowledge exchange space in the event of an epidemic. The NiV outbreak created an opportunity for us to test this theory in real time, allowing us to set up the r/nipah subreddit, a space where people could come together in the time of crisis, be updated regularly with information regarding the virus, and receive advice on their concerns. Our results suggest that Reddit and sites like it can perform as platforms where medical and health information can be distributed, enabling people in need to communicate and discuss any issues encountered during a public health emergency. This could be an extremely efficient use of time, money, and other limited resources during future outbreaks, allowing such forums to deal with minor cases and queries regarding epidemics while leaving valuable professional resources free to deal with more urgent cases.

## Appendix

Multimedia Appendix 1 Spreadsheet of data captured from r/nipah.

## Abbreviations

CDC: US Centers for Disease Control and Prevention
FAQ: frequently asked question
NiV: Nipah virus
PHEIC: public health emergency of international concern
WHO: World Health Organization

## References

[1] (2008). Outbreak Communication Planning Guide.

[2] Glik DC (2007). Risk communication for public health emergencies. Annu Rev Public Health.

[3] Kittler AF, Hobbs J, Volk LA, Kreps GL, Bates DW (2004). The Internet as a vehicle to communicate health information during a public health emergency: a survey analysis involving the anthrax scare of 2001. J Med Internet Res.

[4] Funk S, Gilad E, Watkins C, Jansen VAA (2009). The spread of awareness and its impact on epidemic outbreaks. Proc Natl Acad Sci U S A.

[5] Funk S, Jansen V (2013). The talk of the town: modeling the spread of information and changes in behaviour. Modeling the Interplay Between Human Behavior and the Spread of Infectious Diseases.

[6] Cole J, Watkins C, Kleine D (2016). Health advice from internet discussion forums: how bad is dangerous?. J Med Internet Res.

[7] Cole J Royal Holloway, University of London.

[8] Cole J, Watkins C (2015). International employees' concerns during serious disease outbreaks and the potential impact on business continuity: lessons identified from the 2014-15 West African Ebola outbreak. J Bus Contin Emer Plan.

[9] McInnes C (2018). Old wine in new bottles? Use of twitter by established UK news media during the 2014-15 West African Ebola outbreak. Social Media Use In Crisis and Risk Communication.

[10] Jarvenpaa S, Cantu C, Lim S, Hertel G, Stone DL, Johnson RD, Passmore J (2017). Trust in virtual online environments. The Wiley Blackwell Handbook of the Psychology of the Internet at Work.

[11] Sillence E, Hardy C, Briggs P (2013). Why don't we trust health websites that help us help each other? An analysis of online peer-to-peer healthcare.

[12] Wang Z, Walther JB, Pingree S, Hawkins RP (2008). Health information, credibility, homophily, and influence via the Internet: web sites versus discussion groups. Health Commun.

[13] Chatterjee P (2018). Nipah virus outbreak in India. The Lancet.

[14] (2018). World Health Organization.

[15] Doke O, Kale S, Mujawar FB, More P, More T (2019). A report on Nipah virus. J Drug Delivery Ther.

[16] Chattu VK, Kumar R, Kumary S, Kajal F, David JK (2018). Nipah virus epidemic in southern India and emphasizing. J Family Med Prim Care.

[17] (2018). World Health Organization.

[18] Ariyari S, Vijayan N, Sadanandan R (2018). The first ever Nipah virus outbreak and the best possible response by a tiny state of India. Int J Infect Dis.

[19] (2019). Reddit metrics: r/Kerala.

[20] Alexa topsites.

[21] Rouse M (2016). SearchCIO.com.

[22] Woolley AW, Aggarwal I, Malone TW (2015). Collective intelligence and group performance. Curr Dir Psychol Sci.

[23] r/CSShelp.

[24] r/India.

[25] r/Kerala.

[26] Cole J (2018). Welcome to r/Nipah!.

[27] u/defenceofthedota (2018). Should I get myself checked out for mild fever?.

[28] (2018). WHO: India's Nipah outbreak over.

[29] u/Snowflake1000 (2018). Kerala bids musical farewell to Nipah virus.

[30] u/Snowflake1000 (2019). Disclaimer: research activity on r/nipah.

[31] (2018). World Health Organization.

